# Coupling coordination between higher education and environmental governance: Evidence of western China

**DOI:** 10.1371/journal.pone.0271994

**Published:** 2022-08-22

**Authors:** Yuqing Geng, Fuchang Huang

**Affiliations:** School of Business, Shanghai Dianji University, Shanghai, China; Gonbad Kavous University, ISLAMIC REPUBLIC OF IRAN

## Abstract

Exploring the coupling coordination mechanism between higher education and environmental governance is conducive to understanding the mutual interactions between them and thus enhancing mutual development. This study constructs the coupling coordination mechanism and the aggregated evaluation index system to measure the mutual coordination relations between them, assesses the performances and the coupling coordination degrees between them in the case of western provincial regions in China, and proposes countermeasures based on the prediction results. It is found that the performances of higher education are relatively more stable than those of environmental governance, and the distributions of the average performance grades exist noticeable geographical differences. Besides, the coupling coordination degrees increase with mild fluctuations with relatively small spatial variations, demonstrating the balanced and similar coordinated development status among regions; furthermore, in the future, the gaps in the coordination status among regions will gradually decline. This study highlights the mutual coordination mechanism between the two subsystems, compares the coupling coordination status among regions both temporally and spatially, and proposes specific, generalizable development suggestions that contribute to the academic sector, policymakers, ecology, and sustainability.

## 1. Introduction

Higher education and environmental governance are two critical factors in accelerating social development. The relationship between higher education and environmental governance is complicated [1]; however, it is unclear how they interact and achieve benign and coordinated growth. Previous studies have revealed their complicated relations; on the one hand, higher education can impact environmental governance positively and negatively: better higher education quality will provide better knowledge, skills, human resources, and equipment to enhance environmental governance effectiveness [2–4]; however, the development of the higher education sector will occupy local resources such as annual budgets, land, and human capitals, which will occupy the resources of environmental governance and decrease its performance [5, 6]. On the other hand, environmental governance can impact higher education positively and negatively: environmental governance enhances the local environment, which increases the attractiveness and competitiveness of the local higher education sectors [6, 7]; however, environmental governance requires stricter environmental regulations, which make some laboratories with high environmental pollutions in burden [8]. There are complicated relations between higher education and environmental governance, but how they interact, namely how they coordinate to achieve a benign status, is unclear. Therefore, it is necessary to explore their coupling coordination relations, evaluate the current coordination status, and predict the tendencies. These help stakeholders take corresponding countermeasures to achieve benign and coordinated development between higher education and environmental governance.

In order to explore the coupling coordination development between the two, this article firstly constructs the coupling coordination mechanism between them and sets up the aggregated evaluation index system; secondly assesses the performances and the coupling coordination degree between them with an improved information entropy weight method and coupling coordination model with the case of western provincial regions in China; thirdly predicts the coordination tendencies by using gray prediction model, and proposes suggestions for future coordinated growth.

The contributions are as follows: theoretically, the study constructs the coupling coordination mechanism and the evaluation index system accordingly; practically, differentiated countermeasures are provided based on the temporal-spatial comparisons of the coupling coordination status, which are helpful references to places with similar conditions; sustainably, the study confirms our determination to achieve sustainable development of both society and environment at the same time.

## 2. Literature review

This study defines higher education as the condition and the level where the local post-secondary education sector is qualified and competitive [9, 10]. Higher education evolves by interacting with other systems or factors and can reflect the benefits or problems of other systems (such as the environmental governance system). This study needs to clarify that higher education is not equal to higher education institutions: the latter is only a dimension of higher education. Environmental governance, in this study, is defined as the actions to solve environmental problems and the environmental status brought by the governance actions [11, 12]. Environmental governance in this study mainly focuses on the “public” rather than “individual” actions. It is not precisely equal to environmental quality: the latter is only a dimension of the former. Higher education and environmental governance are two different systems, but they are similar to some degree; for instance, they both depend on human resources and capital devotion. Such differences and similarities also demonstrate that the interactions between the two are complicated and that exploring the coordination between them is significant.

### 2.1. The impact of higher education on environmental governance

Higher education impacts environmental governance positively. Firstly, higher education sectors provide increasing skillful graduates and professional engineers to solve environmental governance problems and enhance environmental governance performance [13, 14]. Besides, better higher education requires more high-qualified teaching and learning regarding environmental governance, which promotes students’ awareness to protect the environment and determination from participating in environmental governance activities [15–17]. Detailed research in some countries has found that expenditure on university lectures benefits environmental governance effectiveness [18]. Moreover, higher education development means more intellectual outputs such as think tanks, advisory boards, patents, industry standards formulation, and research achievements; they provide increasing academic support to accelerate better environmental governance [19, 20]. Many regions have initiated programs and foundations to encourage more outputs of higher education, which directly promote the local environmental governance results [18, 21, 22]. In addition, professional activities accompanied by higher education growth facilitate more local environmental laws and regulations and thus enhance the effectiveness of environmental governance [23, 24].

Higher education also influences environmental governance negatively. The teaching schemes, curriculums, and textbooks accompanied by higher education development are usually relatively lagging compared with the social reality, which to some degree impede the current requirement and the actual performances of environmental governance [25, 26]. Besides, online teaching and learning in higher education sectors are relatively less effective, which decreases the local higher education quality, and makes the students less competitive and skillful in environmental governance practice; thus, environmental governance is negatively affected [27, 28]. At the same time, higher education sectors in some developing regions mainly aim to promote local economic development more than environmental governance when initiating university development plans, limiting local environmental governance [29]. Furthermore, the development of higher education is accompanied by emitting more pollutants and consuming more energy, which are burdens to environmental governance [30, 31]. For example, the growth of higher education in the UK has consumed more energy and produced more pollutants in recent decades, leading to decreased local environmental governance status [32].

### 2.2. The impact of environmental governance on higher education

Environmental governance positively impacts higher education. Firstly, benign environmental governance results provide better environmental resources such as good lands, clear water, and fresh air, which are competitive factors to attract more talented teachers and students and thus enhance the local higher education quality [33]. Meanwhile, more effective environmental governance practices stimulate technology innovation and discipline development (such as environmental science and engineering, management science and engineering). These enhance the skills, performances, and competitiveness of university professionals or specialists and further improve the quality of the local higher education sector [34, 35]. Moreover, better environmental governance status benefits the college students both physically and emotionally, increases their recognition of green campuses, promotes better study quality, and thus enhances the quality of the local higher education [36, 37]. Furthermore, as a typical teaching mode, environmental governance-related practices promote students’ environmental awareness and optimize their environmental protection behaviors; as typical examples, environmental governance-related cases enhance teaching effectiveness: these contribute to better outputs or performances of the local higher education sector [6, 38, 39].

Environmental governance also affects higher education negatively. First, environmental governance occupies much public funding; when the local budgets or funds are constant, environmental governance means more funding will be allocated, and less money will be given to the local higher education sector, which limits the development of the local higher education sector [40]. Besides, environmental governance sometimes requires volunteering participation of college students, which means that students are more likely to spend less time in the study within the limited time, their competitiveness will decrease, and the performance of the local higher education sector will decline [41–43]. In addition, stricter environmental governance requires laboratories in universities to renew more environment-friendly equipment, which is a fiscal burden for some laboratories that are not well funded, and thus hinders the research outputs of the laboratories and reduces the development level of the higher education in the short term [8, 44, 45].

### 2.3. The interactions between higher education and environmental governance

Previous studies have proved that higher education and environmental governance interact complicatedly; therefore, it is needed to explore their interaction mechanism so that mutual positive interactions and balanced growth can be achieved. Coupling coordination, which describes the interaction status between systems, can evaluate the complicated interactions between higher education and environmental governance. The coupling coordination model, which reflects the interactions from disharmony to harmony between systems, has proved valuable and appropriate for analyzing systems’ mutual effects via interactions [46, 47]. Previous studies have put the coupling coordination model into practice to analyze the coupling coordination status between environmental governance and other social systems (such as tourism, social economy, and urbanization) and analyze the coupling coordination status of dimensions of higher education [48–51]. These studies prove that assessing the interactions between higher education and environmental governance with the coupling coordination model is necessary and applicable. However, there are limited studies to explore the coupling coordination interactions between higher education and environmental governance from both the temporal and spatial perspectives; therefore, the proposed suggestions for different places to enhance the coordinated growth between higher education and environmental governance are somehow less convincing.

It is necessary to analyze the coupling coordination status between higher education and environmental governance, and deepen the understanding of such interactions. It benefits more effective evaluation of higher education and environmental governance and helps propose more suitable suggestions for coordinated growth between them. Nevertheless, empirical research on the coupling coordination between them is not easy because there are no precise coupling coordination mechanisms and no widely accepted theoretical frameworks or indicators to measure them. Additionally, though conducive to this study, former studies regarding the relations of higher education and environmental governance usually ignore spatial-temporal comparisons of multiple places, which is essential to understand the coordination relations between them comprehensively. Therefore, in this study, it is necessary to conduct the coupling coordination research between higher education and environmental governance by comparing multiple places from both temporal and spatial perspectives.

### 2.4. Research area of this study

**Fig 1:**
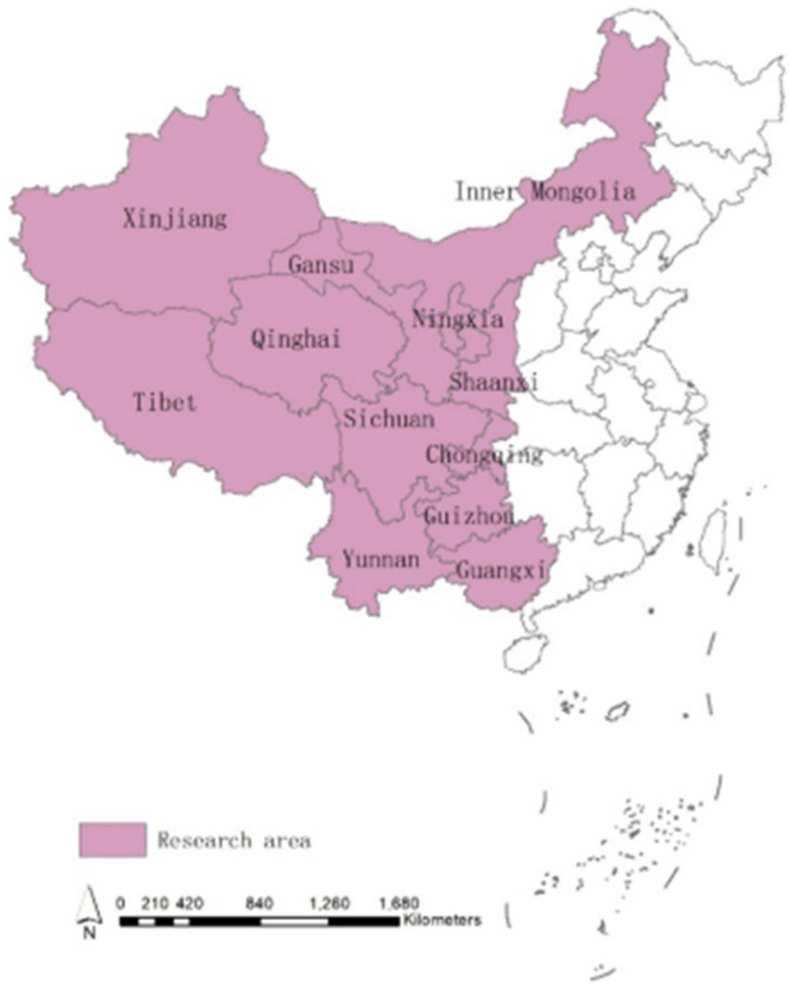
Research area of this study.

This study selects the 12 western provincial regions of the West China Development Strategy, including Shaanxi, Inner Mongolia, Ningxia, Gansu, Qinghai, Xinjiang, Tibet, Chongqing, Sichuan, Guizhou, Yunnan, and Guangxi (Fig 1). We select the 12 western provincial regions due to their representativeness: regarded as the lagging or less developed places traditionally, and benefiting from the West China Development Strategy in the recent decades, these regions have witnessed fast growth in higher education and increasing efforts of environmental governance from the authorities; however, these regions still share apparent differences such as the imbalanced growth level in higher education and uneven devotion to environmental governance, which makes it significant to understand the coordination interactions between higher education and environmental governance in these places. Comparing the western regions in China contributes to the adequate understanding of the intricate coordination between higher education and environmental governance and the proposal of more specific meanwhile applicable countermeasures to enhance coordinated mutual growth. These suggestions are also good references to others facing similar conditions.

## 3. Materials and methods

### 3.1. Coupling coordination mechanism

**Fig 2:**
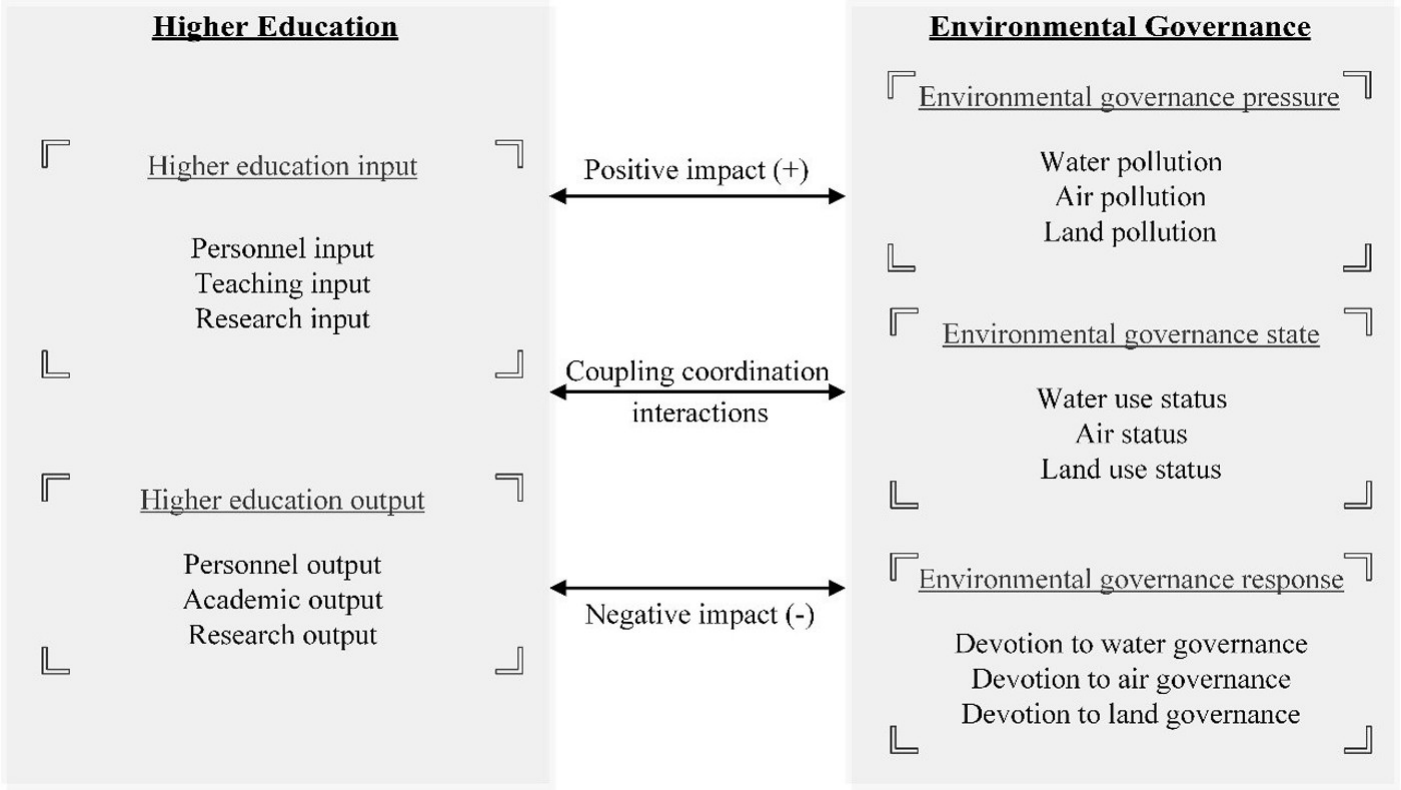
Coupling coordination mechanism.

There are bilateral coupling coordination interactions between higher education and environmental governance; therefore, it is possible to construct a coupling coordination mechanism to illustrate such complicated coordination relations (Fig 2).

In this mechanism, the higher education subsystem and the environmental governance subsystem follow the “input-output” framework and “pressure-state-response” frameworks, respectively [9, 50, 52].

For the higher education subsystem: (1) higher education input (measuring the input quality or level of personnel, teaching, and research) positively or negatively affect the three dimensions of environmental governance. For instance, more input to teaching leads to better performances of students and higher awareness to participate in the environmental governance activities; the more input to research in environmental governance, the higher the possibility of applying the more advanced technologies to reduce environmental pollution and achieve better environmental governance performances. On the contrary, more resources or funding allocated to the teaching and research of the local higher education sector may occupy the resources or funding of environmental governance, which to a certain degree restricts the effectiveness of the responsive actions of environmental governance. (2) Higher education output (measuring the output quality or level of personnel, teaching, and research) impacts environmental governance positively and negatively. Specifically, more high-qualified personnel output provides academic support to reduce environmental waste, enhance environmental governance states, process environmental impact assessment, and take responsive actions to treat environmental governance issues. The high-quality academic or research outputs provide theoretical foundations or guidelines to enhance the environmental governance status and offer resources, benefits, policies, and countermeasures to decrease environmental governance pressure. On the contrary, the research output of higher education (such as the byproducts of chemical experiments like wastewater and residue) directly pollutes the environment, decreases the environment quality, and increases the burden of environmental governance response; some controversial or even unethical academic output is likely to decrease the state of the environment and hinder the quality of environmental governance responsive actions [3, 5, 6, 45, 53].

For the environmental governance subsystem: (1) environmental governance pressure (measuring the water, air, and land pollution) affects the input and output of higher education in positive and negative directions. In detail, increasing environmental pollution leads to increased research funding to study environmental governance. As a result, the research output in this field will increase accordingly. On the contrary, severe environmental governance problems such as pollution are detrimental to the persons in the higher education sector, who affect the input and output quality of higher education; also, the increasing pressure of environmental governance decreases the attractiveness and competitiveness of the local higher education and thus hinders the personnel input and output quality of higher education. (2) Environmental governance states (measuring the average water, air, and land status) can promote or impede the input and output of higher education. For example, improved environmental governance states enhance the quality of higher education input: improved environment quality provides significant references for researchers exploring environmental-related issues such as sustainability and carbon neutralization and provides proper cases for teachers to discuss environmental governance. Besides, the enhanced environmental status improves higher education output: benign environment status enhances the satisfaction and performances of participants in higher education. On the contrary, deteriorating environmental quality sometimes restricts higher education: capital investments in higher education are more likely to escape from places with poor environmental quality; thus, both the input and output of higher education will be impeded. (3) Environmental governance response (measuring the reactive quality to treat water, air, and land) supports and restricts higher education. In detail, more fiscal devotions of environmental governance treatment facilities encourage higher education personnel to focus on this field and pay more attention to developing related facilities, which in turn facilitates the growth of personnel and their high-qualified performances in academics and research. Besides, laws and regulations aiming to enhance environmental governance encourage professionals in the higher education sector to devote more resources to study and propose corresponding countermeasures. On the contrary, as the total amount is limited, investments in environmental governance practices occupy the input to higher education, which hinders the high-quality growth of higher education [9, 54–58].

### 3.2. Evaluation index system

This article constructs an aggregated evaluation index system based on the coupling coordination mechanism to empirically assess higher education and environmental governance’s performances and coupling coordination degrees. The index should meet the following principles: (1) the index should cover the primary contents and main dimensions of higher education and environmental governance; (2) the index should be accepted and applied in previous research and have been proved scientifically; (3) the index can measure and reflect the mutual relationship between these two subsystems; (4) the data should be temporally and spatially available; (5) the index should be easy to understand [55, 56]. After literature referring, expert interviews, qualitative analysis, and significance tests, indices are screened and selected, with the details in Table 1, where + means this is a positive index, and–is a negative one.

**Table 1:** Evaluation index system of the coupling coordination mechanism.

Subsystem	Dimension	Indices	Source
Higher education	Input	Proportion of faculty in the total population (+)	[49]
		Student-teacher ratio (-)	[49, 51]
		Education funding input per student (+)	[49, 51]
		Research funding input per faculty (+)	[49]
	Output	Proportion of students of higher education in the total population (+)	[9, 49]
		Number of academic papers per higher education personnel (+)	[57]
		Number of publications per higher education personnel (+)	[57]
		Number of research projects per higher education personnel (+)	[57]
Environmental governance	Pressure	Wastewater emissions per capita (-)	[58, 59]
		Dust emissions per capita (-)	[60, 61]
		Sulfide emissions per capita (-)	[47, 60]
		Solid waste emissions per capita (-)	[50, 58]
	State	Volume of water use per capita (-)	[59, 60]
		Average concentrations of NO_2_ (-)	[47, 62]
		Average concentrations of SO_2_ (-)	[47, 62]
		Average concentrations of PM_10_ (-)	[47, 62]
		Forest cover rate (+)	[63, 64]
	Response	Proportion of investment in water treatment in financial expenditure (+)	[59, 65]
		Proportion of investment in air treatment in financial expenditure (+)	[65, 61]
		Proportion of investment in solid waste treatment in financial expenditure (+)	[65, 50]
		Annual number of approved environmental impact assessment documents (+)	[50]

In the evaluation index system, the subsystem of higher education is sub-divided into two dimensions with eight aggregated indices, which are based on the input-output framework [9, 53]. The dimension of higher education input is represented by the human and financial input from the perspectives of teachers and students during higher education sector development. In detail, the two indices, “proportion of faculty of higher education in the total population” and “student-teacher ratio in higher education,” reflect the human capital input quality to both teachers and students; the two indices, “education funding input per student in higher education” and “research funding input per faculty in higher education” reflect the monetary input quality to teachers and students. The dimension of higher education output is represented by the teachers’ and students’ performance quality. In detail, the index “proportion of students of higher education in the total population” reflects the output quality from the students’ perspective; the indices “number of academic papers per higher education personnel” and “number of publications (except academic papers) per higher education personnel” reflect the output quality of academics from the teachers’ perspective; the index “number of research projects per higher education personnel” reflects the output quality of research from the teachers’ perspective. These indices mainly use the proportion and ratio of relevant stakeholders to assess higher education growth because it is more proper to reduce absolute quantity differences and to reflect the development level based on the local population size.

The subsystem of environmental governance is sub-divided into three dimensions with 13 indices based on the Pressure-State-Response framework [27, 66]. Environmental governance pressure is the pressure on or pollution of natural resources or the environment imposed by human activities. The pressure happens to water, air, and land; thus, four indices are selected to demonstrate the pressure of environmental governance on these aspects. In specific, the indices “wastewater emissions per capita” and “solid waste emissions per capita” demonstrate the pressure on environmental governance from water and land perspectives; the indices “dust emissions per capita” and “sulfide emissions per capita” demonstrate the pressure from the air perspective. The dimension of the environmental governance state represents the current situations of environmental governance from the perspectives of water, air, and land. In specific, there are five indices: the index “volume of water-use per capita” illustrates the state of water use; the indices “average concentrations of NO2, SO2, and PM10” illustrate the state of air; the index “forest cover rate” illustrates the quality state of the land. The environmental governance response dimension demonstrates the society’s action to change environmental problems. Four indices, namely “proportion of investment in water treatment in financial expenditure,” “proportion of investment in air treatment in financial expenditure,” “proportion of investment in solid waste treatment in financial expenditure,” and “the annual number of approved environmental impact assessment documents,” depict how society responds to environmental governance efficiently from both public finance and policy perspectives.

### 3.3. Calculation

When assessing the performances and coupling coordination degrees, weights of indices will be first determined. Several methods calculate the weights of indices and the systems’ performances. One valuable method is the improved information entropy weight method, which combines the traditional information entropy weight method and the technique for order preference by similarity to an ideal solution method (TOPSIS). There are several advantages of the improved information entropy weight method, including (1) easy to calculate, (2) getting weight objectively without factitious manipulations, (3) exhibiting relatively importance of different indices and making relative variations much clearer, and (4) covering deficiencies of using one method solely such as critical components missing [66, 67]. This article thus chooses the improved information entropy weight method to do relevant calculations. While it is not new, it is seldom used in the coordination measurement between higher education and environmental governance, which is innovative to some degree.

The data collection process is mainly from the China Statistical Yearbook on Environment, China Statistical Yearbook on Education, Compilation of Statistics on Science and Technology of Higher Education Institution, and China Statistical Yearbook (2005–2020). These references are from national authorities and guarantee the objectivity and correctness of the research results.

#### 3.3.1. Performance of subsystems

1. Standardize the matrix *X*. For the original matrix *X*, *i* and *j* are the option and index, respectively. This article selects the data of 12 western provincial regions enjoying the West China Development Strategy for 16 years; thus, there are 12×16 = 192 options in total for every index. According to Table 1, there are 9 indices in the higher education subsystem and 13 indices in the environmental governance subsystem. Formula (1) is for negative indices, and formula (2) is for positive ones. Besides, *i* = 1, 2, …, m; *j* = 1, 2, …, n. In order to avoid value insignificance, values of 0 in the original matrix are replaced as 0.01, which is common in the standardization process [68, 69]. The new matrix after standardization is defined as *X*′.
$${X^{\prime }}_{ij}=1-\frac{X_{ij}}{\sum_{i=1}^{n}X_{ij}}$$ (1)
$${X^{\prime }}_{ij}=\frac{X_{ij}}{\sum_{i=1}^{n}X_{ij}}$$ (2)
2. Calculate *ln f*_*ij*_ to avoid insignificance, and *Y*_*j*_, the information entropy of the index *j*, given $X^{\prime }={\left(X_{ij}^{\prime }\right)}_{mn}$.
$$f_{ij}=\frac{1+X_{ij}^{'}}{\sum_{i=1}^{m}\left(1+X_{ij}^{'}\right)}$$ (3)
$$Y_{j}=-\left(\overset{m}{\underset{i=1}{\sum }}f_{ij}\;ln\;fij\right)$$ (4)
3. Calculate *T*_*j*_, the weight of the index *j*.
$$T_{j}=\frac{1-Y_{j}}{n-\sum_{j=1}^{n}Y_{j}}$$ (5)
4. Then we determine *X*″ as the standardized matrix of a given year; *i*′ and *j*′ are the option and index of the given year, respectively; there are 12 provincial regions; thus, there are 12 options in total in a given year; besides, *i′* = 1, 2, …, m; *j′* = 1, 2, …, n. We can get $\underset{1\leq j^{\prime }\leq n}{max}X_{i^{\prime }j^{\prime }}^{''}$ and $\underset{1\leq j^{\prime }\leq n}{min}X_{i^{\prime }j^{\prime }}^{''}$, the maximized and the minimized values of the standardized matrix *X*″, respectively. Calculate the standardized matrix’s ideal positive and negative values in a given year *X*″ with Formula 6 and 7, respectively.
$$X_{j^{\prime }}^{\text{''}+}=\left(\underset{1\leq j^{\prime }\leq n}{max}X_{i^{\prime }1}^{''},\underset{1\leq j^{\prime }\leq n}{max}X_{i^{\prime }2}^{''},\ldots ,\underset{1\leq j^{\prime }\leq n}{max}X_{i^{\prime }n}^{''}\right)$$ (6)
$$X_{j^{\prime }}^{\text{''}-}=\left(\underset{1\leq j^{\prime }\leq n}{min}X_{i^{\prime }1}^{''},\underset{1\leq j^{\prime }\leq n}{min}X_{i^{\prime }2}^{''},\ldots ,\underset{1\leq j^{\prime }\leq n}{min}X_{i^{\prime }n}^{''}\right)$$ (7)
5. Calculate the distance value of the option *i*′ to the ideal positive value ($V_{i^{\prime }}{}^{+}$) and the distance value of the option *i*′ to the ideal negative value ($V_{i^{\prime }}{}^{-}$).
$${V_{i^{\prime }}}^{+}=\sqrt{\overset{n}{\underset{j=1;j^{\prime }=1}{\sum }}T_{j}{\left(X_{i^{\prime }j^{\prime }}^{''}-X_{j^{\prime }}^{\text{''}+}\right)}^{2}}$$ (8)
$${V_{i^{\prime }}}^{-}=\sqrt{\overset{n}{\underset{j=1;j^{\prime }=1}{\sum }}T_{j}{\left(X_{i^{\prime }j^{\prime }}^{''}-X_{j^{\prime }}^{\text{''}-}\right)}^{2}}$$ (9)
6. Calculate $PER_{i^{\prime }}$, the performance of the option *i*′ in the subsystem.
$$PER_{i^{\prime }}=\frac{{V_{i^{\prime }}}^{-}}{{V_{i^{\prime }}}^{+}+{V_{i^{\prime }}}^{-}}$$ (10)

Then we can set up the evaluation grades for the performance. The performance value fluctuates between 0 and 1; thus, this article divides the performance into five grades based on the equalization principle, commonly used in previous research [69]. There are Grade Poor (0.0000–0.1999), Ordinary (0.2000–0.3999), Fair (0.4000–0.5999), Good (0.6000–0.7999), and Excellent (0.8000–1.0000).

#### 3.3.2. Coupling coordination degree

This article uses the coupling coordination model to assess the coupling coordination degree between higher education and environmental governance. The details are as follows.

1. Calculate coupling degree (Z). *PER*_*he*_ and *PER*_*eg*_ are the performance of higher education and environmental governance in a given year, respectively.
$$\text{Z}={\left\{\frac{PER_{he}\times PER_{eg}}{{\left(\frac{PER_{he}+PER_{eg}}{2}\right)}^{2}}\right\}}^{\frac{1}{2}}$$ (11)
2. Calculate the coupling coordination degree (COU) of a given year. ρ and λ are coefficients, as higher education and environmental governance have the same importance in the coupling coordination mechanism, and the sum of these two coefficients is 1; thus, both coefficients are the same, equal to 0.5 [70].
$$\text{COU}=\sqrt{\text{Z}\times \left(\rho PER_{he}+\lambda PER_{eg}\right)}$$ (12)

Then we can set up the evaluation grades for the coupling coordination degree (COU). The values of the grades are between 0 to 1; thus, this article divides COU into ten grades with equal intervals, which is also common in previous research [69]. The details are in Table 2.

**Table 2:** Evaluation grades for COU.

Value of COU	Grade	Category
0.0000–0.0999	High incoordination	Adverse coordination
0.1000–0.1999	Serious incoordination	
0.2000–0.2999	Moderate incoordination	
0.3000–0.3999	Slight incoordination	
0.4000–0.4999	Approaching incoordination	Transitioning coordination
0.5000–0.5999	Approaching coordination	
0.6000–0.6999	Slight coordination	Benign coordination
0.7000–0.7999	Moderate coordination	
0.8000–0.8999	Favorable coordination	
0.9000–1.0000	high coordination	

#### 3.3.3. Prediction of coupling coordination degree

This article uses the gray prediction GM (1,1) model to predict the tendency of COU. This model applies especially when the sample is limited with data [71]. GM(1,1) model is a widely used method, but it is new in measuring the coordination status between higher education and environmental governance. The details are as follows.

1. In the original times series *COU*_0_ = {*COU*_0_(1), *COU*_0_2,…, *COU*_0_ (*n*)}, the time sequence starts from 1 to *n*. In this article, the data are selected for 16 years, thus *n* = 16. The new sequence *COU*_1_ = {*COU*_1_(1), *COU*_1_2,…, *COU*_1_(*n*)} will be generated based on $COU_{1}\left(t\right)=\sum_{i=0}^{t}COU_{0}\left(i\right)$, then, calculate the differential equation with formula 13. μ here is development grey value; *α* here is endogenous control value. Here *t* = 1,2, …, n.
$$\mu =\frac{dCOU_{1}\left(t\right)}{dt}+\alpha COU_{1}\left(t\right)$$ (13)
2. *A* = [-*Z*_1_(2), -*Z*_1_(3),…, -*Z*_1_(*n*),1,1,1,…,1]^*T*^ and Y = [*COU*_0_(2), *COU*_0_(3),…, *COU*_0_(*n*)]^*T*^; then calculate $\hat{a}={\left(A^{T}A\right)}^{-1}A^{T}Y$ and get the cumulative sequence prediction model $\hat{COU}\left(t+1\right)=\left[COU_{0}\left(1\right)-\frac{\mu }{\alpha }\right]e^{-\alpha t}+\frac{\mu }{\alpha }$. The sequence is proper and acceptable to predict the tendencies if a is no greater than 0.5 [66]. Besides, in order to enhance the prediction accuracy, we can obtain the residual difference ${\text{RD}}_{0}\left(t\right)=COU_{0}\left(t\right)-\hat{COU}\left(t\right)$, relative error $\text{RE}\left(\text{t}\right)=\frac{{\text{RD}}_{0}\left(t\right)}{COU_{0}\left(t\right)}\times 100\text{\%}$, and small error probability $EP=p\left(\left|{\text{RD}}_{0}\left(t\right)-{\bar{RD}}_{0}\right|<0.6745V_{COU}\right)$, where $V_{\text{COU}}^{2}=\frac{1}{n-1}\sum_{t=2}^{n}{\left({\text{COU}}_{0}\left(t\right)-{\bar{COU}}_{0}\right)}^{2}$ and ${\bar{COU}}_{0}=\frac{1}{n-1}\sum_{t=2}^{n}{\text{COU}}_{0}\left(t\right)$ are the variance and mean of COU_0_(*t*), and $V_{\text{RD}}^{2}=\frac{1}{n-1}\sum_{t=2}^{n}{\left({\text{RD}}_{0}\left(t\right)-{\bar{RD}}_{0}\right)}^{2}$ and ${\bar{RD}}_{0}=\frac{1}{n-1}\sum_{t=2}^{n}{\text{RD}}_{0}\left(t\right)$ are the variance and mean of RD_0_(*t*). If EP is no less than 0.6, the sequence is applicable for predictions [70].

## 4. Results and discussions

### 4.1 Performance analysis temporally and spatially

**Fig 3:**
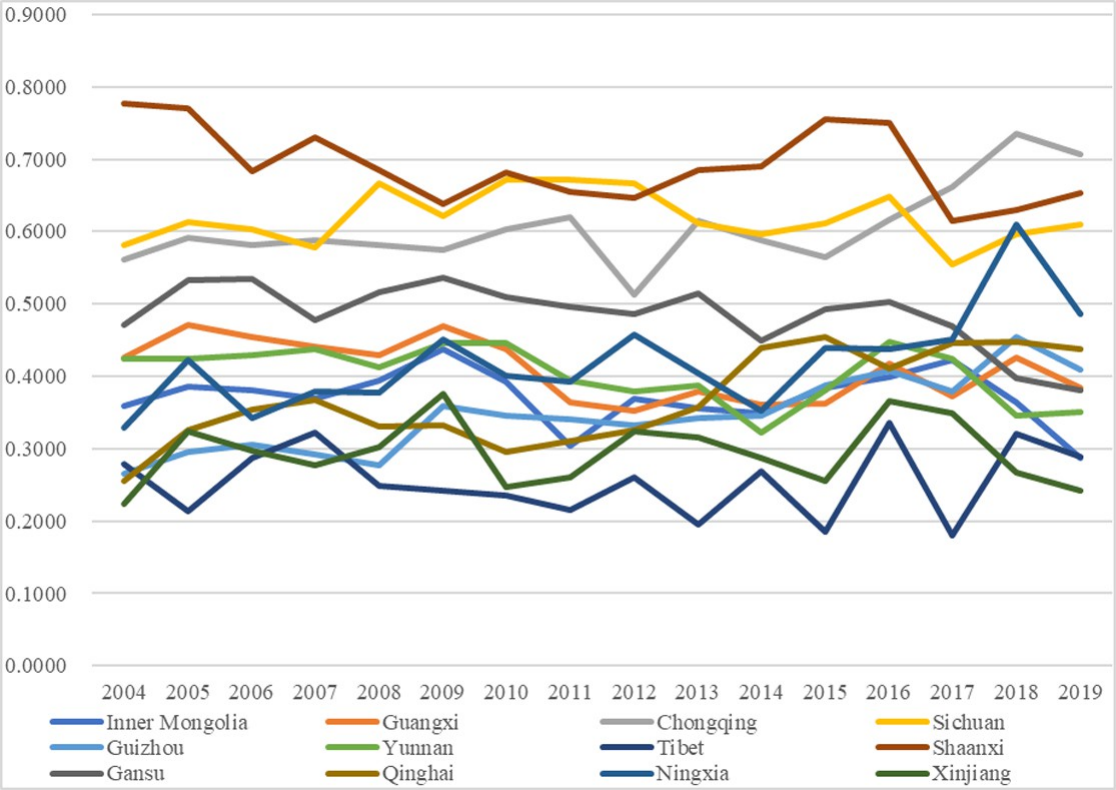
Performance of the higher education subsystem.

**Fig 4:**
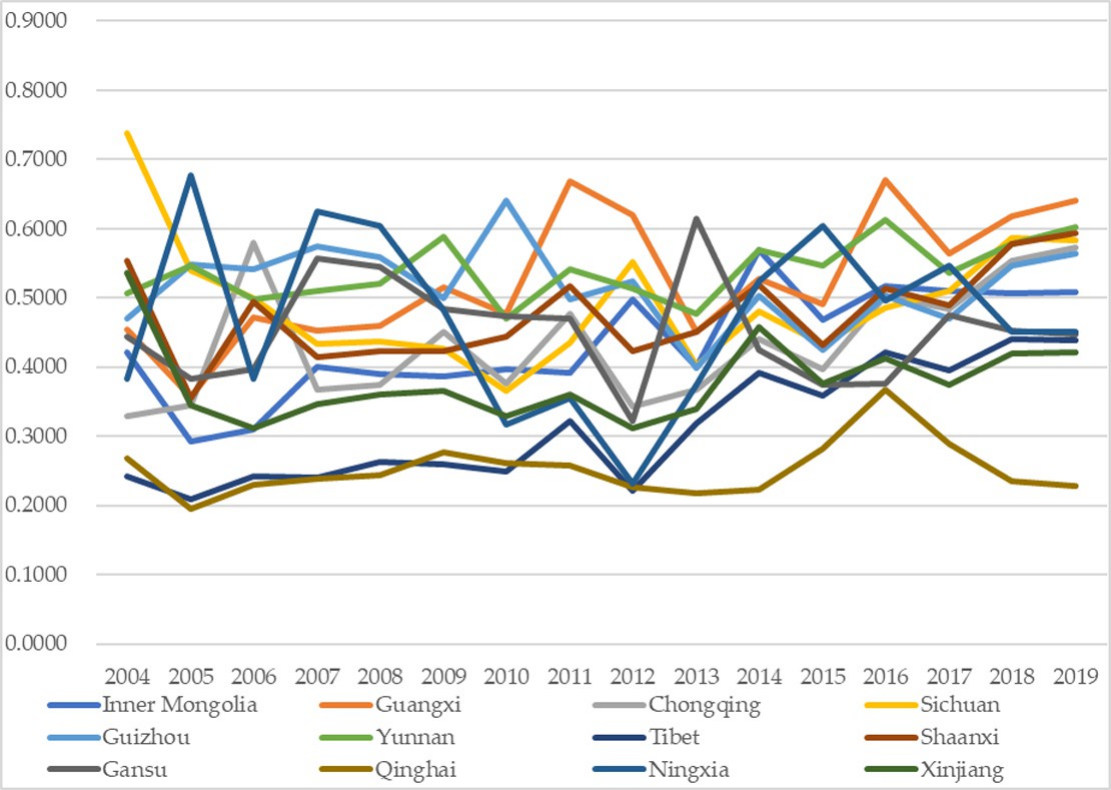
Performance of the environmental governance subsystem.

Figs 3 and 4 exhibit the temporal changes in the performances of higher education and environmental governance, respectively, with the exact values shown in the appendix S1 and S2 Tables. Comparing the two subsystems, we can find that their performances exhibit differences with some other interesting findings.

1. The performances of the higher education subsystem were relatively more stable than those of the environmental governance subsystem. In detail, most regions fluctuated within the same grades or two adjacent grades. For instance, Shaanxi fluctuated within the grade Good (0.6000–0.7999), and Xinjiang fluctuated within the grade Ordinary (0.2000–0.3999); Chongqing and Sichuan fluctuated between Fair (0.4000–0.5999) and Good (0.6000–0.7999), Guangxi, Yunnan, Inner Mongolia, and Qinghai fluctuated between Ordinary (0.2000–0.3999) and Fair and Tibet fluctuated between Poor (0.0000–0.1999) and Ordinary. The relatively stable performances of higher education were mainly because of the annual continuous and stable investments in higher education from the local authorities. However, there were some fluctuations in specific years for some regions, which was mainly because of the hysteresis effect of the higher education outputs such as academic papers and publications, which led to imbalanced performances between the dimensions of higher education input and higher education output, and which led to fluctuating performances of higher education among years. The relatively mild fluctuations of higher education are also proved by other cases or regions in other literature [49]; such similarity demonstrates that it is possible to maintain the higher education level at a relatively stable condition by persistently inputting resources and encouraging continuous output.
2. The performances of the subsystem of environmental governance were relatively more fluctuating; in other words, many regions fluctuated among the three grades. In specific, within the five grades, namely Grade Poor (0.0000–0.1999), Ordinary (0.2000–0.3999), Fair (0.4000–0.5999), Good (0.6000–0.7999), and Excellent (0.8000–1.0000), Guangxi, Sichuan, Guizhou, and Ningxia (one-third of the regions) fluctuated among grades Ordinary, Fair and Good. Besides, the other two-thirds of the regions fluctuated between two grades, and no one fluctuated within the exact grade, just like the performances of the higher education subsystem. Moreover, there were relatively more obvious upgrading tendencies for the performances. In detail, from 2004 to 2019, 9 of the 12 regions witnessed increases in performance values, among which five regions (Guangxi, Chongqing, Yunnan, Tibet, and Ningxia; almost half the regions) had upgradations to higher grades, which was more than the higher education subsystem (7 regions had increase and four regions had upgradations). The more apparent fluctuations of the environmental governance performances were mainly because of the imbalanced investment differences in waste treatments per capita among different years, which indirectly proves the significant effects of fiscal investments from local authorities on effective environmental governance. A former study also proves the annual variations of performances in environmental governance [65], demonstrating that there are still differences or gaps in environmental governance among years in these regions, and it is needed to take action to balance the investment differences among regions and years and to achieve better performances.

**Fig 5:**
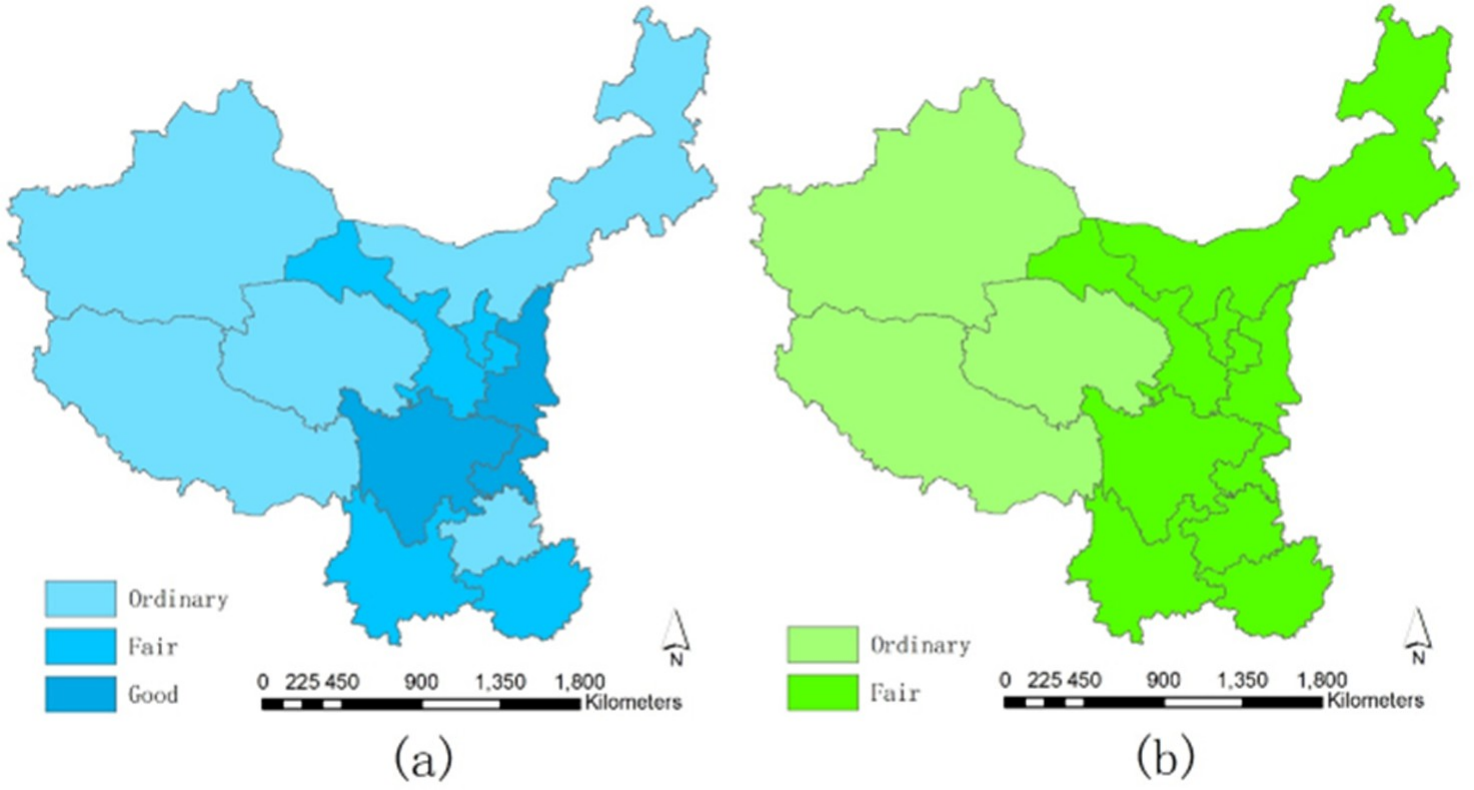
Average grades of performances. (a) Higher Education Subsystem; (b) Environmental Governance Subsystem.

Fig 5 exhibits the spatial distributions of both subsystems’ average performance grades. Comparing the average grades of the two subsystems, we can see some similarities and differences, which are new and exciting findings. More details are as follows.

1. The spatial distributions of the average performance grades of the two subsystems are similar in that the West regions had weaker performances than other regions. Xinjiang, Tibet, and Qinghai had lower grades than others in the higher education and environmental governance subsystem, demonstrating higher education’s relative input and output, and the relative pressure, status, and response to environmental governance were less satisfying. Former studies evaluated the actual performances of these three regions’ higher education and environmental governance and showed similar results [48, 58, 65], proving these regions lack sufficient development in higher education and environmental governance. Thus, these regions should carefully introspect the current weakness and gradually achieve benign growth; besides, the national authority should pay more attention to these western regions, introduce more targeted development measures and invest more funds and resources to accelerate the development of both higher education and environmental governance in these regions.
2. The spatial distributions of each subsystem exhibited a noticeable difference: there were relatively significant regional differences in the average performances for the higher education subsystem, whereas the spatial differences of the environmental governance subsystem were slight. In detail, for the higher education subsystem, there were also two regions falling in the Ordinary grade in the north and south respectively (Inner Mongolia and Guizhou) except for the three western regions mentioned before; besides, there were three regions (Sichuan, Chongqing, and Shaanxi) in the Good grade, which further expanded the spatial imbalances and differences. Such variations demonstrated that the traditional advanced regions in higher education (Sichuan, Chongqing, and Shaanxi) still maintained relatively high competitiveness and benign performances. On the contrary, for the environmental governance subsystem, except for the three western regions, all the other regions were in the same Fair grade; the minor spatial differences demonstrated that the environmental governance performances among regions were similar. Comparing the two subsystems, we could interpret that the regions with relatively low performances in higher education can achieve relatively high environmental governance (Inner Mongolia and Guizhou). Such results are somehow inconsistent with the former studies, which believe that regions in western China are almost weak in higher education, and the environmental governance among regions is not much balanced [72, 73]. Such differences are mainly because this study selects indicators from the perspective of "per capita," thus reducing the performance differences caused by significant population variations and obtaining more accurate results. Such innovative findings provide a valuable reference to the authority that future policies should consider population differences of regions in order to achieve better performances in higher education and environmental governance.

### 4.2 Coupling coordination degree (COU) analysis temporally and spatially

**Fig 6:**
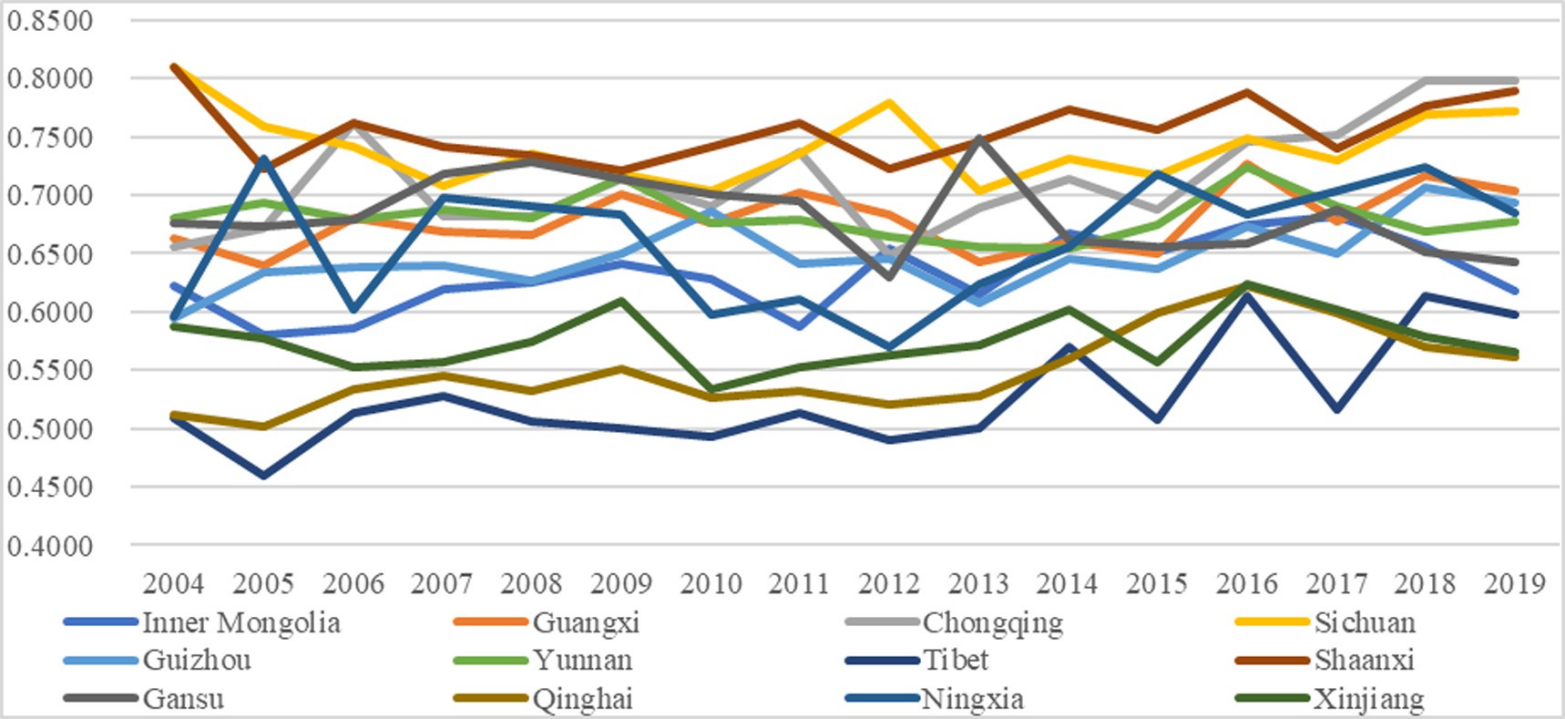
Temporal changes of COU.

Fig 6 exhibits the temporal changes of COU with the exact values shown in S3 Table. There are some interesting findings.

1. We can find that most regions witnessed mild fluctuations of COU. In detail, some regions had apparent fluctuations; for instance, Gansu in 2013 had an apparent increase, and Tibet in 2015 and 2017 faced an evident decline; such noticeable fluctuations were mainly because of the apparent changes in specific subsystems: Gansu in 2013 had an apparent increase in environmental governance performance, whereas Tibet in 2015 and 2017 had apparent declines in higher education performances. Such fluctuations illustrate that specific subsystems would promote or impede the coupling coordination relations in the coupling coordination mechanism. This finding tells policymakers that they should make specific policies and take specific actions to decrease the impeding subsystems’ effects and gradually achieve more benign coordination between subsystems. The fluctuations are common in the coupling coordination relations, which are also seen in previous studies, such as between tourism and air environment governance [46]; thus, we do not have to worry too much about the coupling coordination fluctuations, as long as we take enough practical approaches to keep COU in the positive directions.
2. Another finding is that almost all regions fluctuated between the approaching and moderate coordination grades within the years. The regions fluctuated among the three grades, illustrating that the differences in COU were relatively small, and regions had relatively balanced and similar coordinated development statuses. Such relatively few differences demonstrate that all regions achieve similar efficient coordination growth, which proves that it is possible to reduce the statistical bias caused by demographical and geographical differences and to depict more accurate results when we compare regions from the perspectives of "per capita" or "proportion" rather than the "absolute quantity." Such results also prove the success of the high-quality development strategy in China, which aims to narrow the gaps among regions and achieve more benign and coordinated growth in every aspect. Certain studies use the "absolute quantity" indicators to evaluate the coupling coordination development of other systems (such as environmental protection and urbanization) and obtained similar mild fluctuations and few differences. That is mainly because the regional differences are not so apparent (the absolute quantity differences are less obvious and are similar to "per capita" numbers) [52, 63]. The comparisons provide references to policymakers that "per capita" or "proportion" should be carefully considered when taking measures to achieve better coupling coordination between systems.

**Fig 7:**
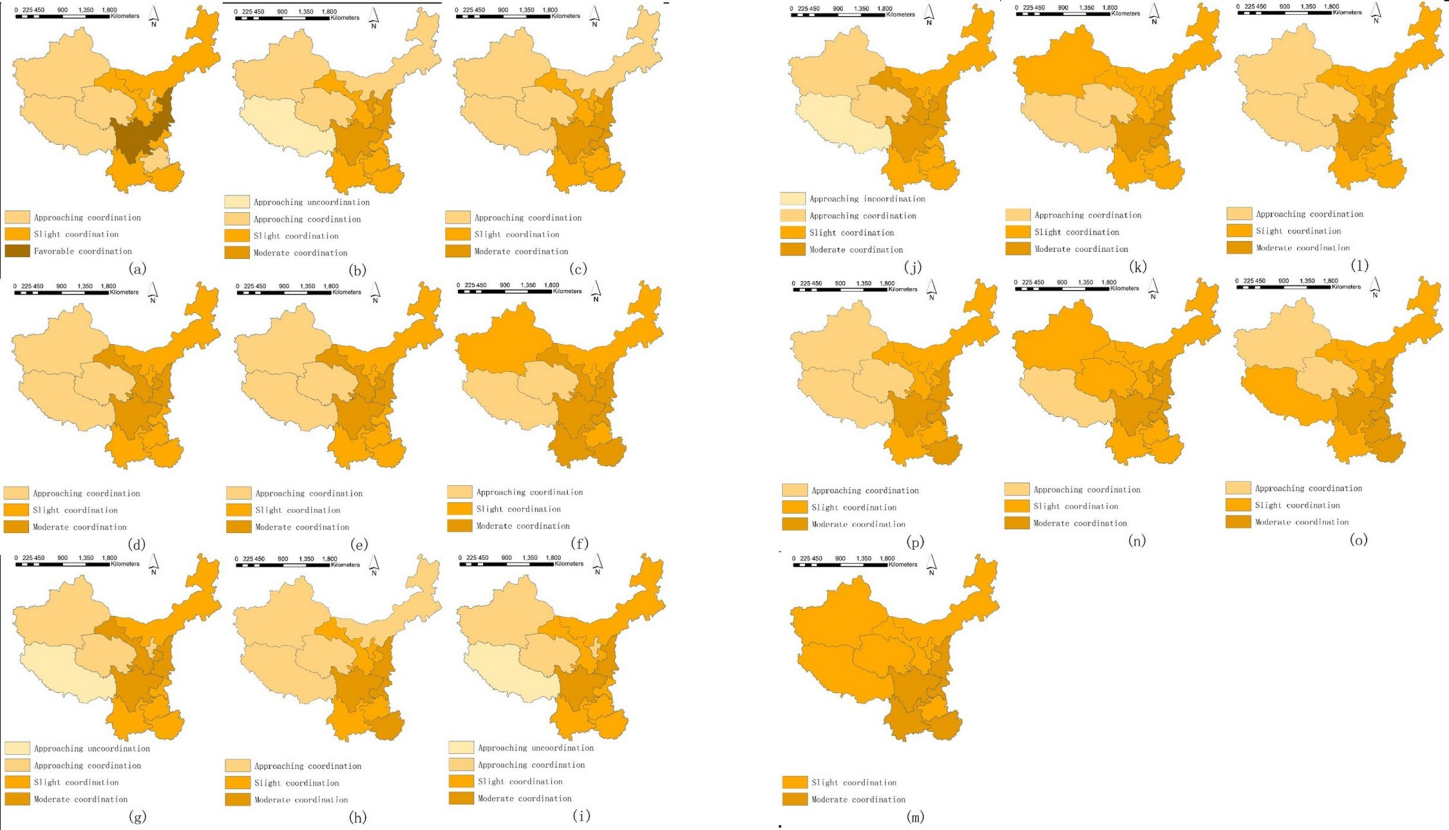
**a.** Spatial variations of COU. (a-i) 2004–2012. **b.** Spatial variations of COU. (j-p) 2013–2019.

The spatial variations of the coupling coordination relations between higher education and environmental governance are in Fig 7A and 7B. Some findings are as follows.

1. Generally, the grades of COU were upgrading in the observed years. For instance, Ningxia and Guizhou upgraded their grades from approaching coordination in 2004 to slight coordination in 2019; Guangxi and Chongqing upgraded from slight coordination in 2004 to moderate and favorable coordination in 2019, respectively. The upgrades prove that these regions can attain a more benign coordination status between higher education and environmental governance when enjoying preferential policies such as the high-quality and develop-the-west strategies. In other studies, we can also find that taking China as an example, the coupling coordination status of other systems, such as finance and air environment, is upgrading [70], illustrating the possibility of realizing harmonious, coordinated development between systems. Thus, the authority should continuously introduce incentives to encourage coordinated development in the western regions.
2. The gaps of COU between the regions in the west and others were gradually declining. In detail, in 2005, the three regions in the west (Xinjiang, Qinghai, and Tibet) were in the approaching coordination and approaching incoordination grades, whereas, since 2017, these regions had reached the higher grade of slight coordination, which gradually declined their gaps with other regions. Such decline in gaps was mainly because of the tremendous inputs and investments in higher education and environmental governance in these three regions from the national and local authorities. The three regions enjoyed tremendous benefits from policies such as the West China Development Strategy, the West Special Program of National Social Science Foundations of China, the Plan of Pair Support for Higher Education Institutions in the Western Regions, the River Manager System, the Policy of Constructing Modern Environmental Governance System, and Liangshan Development (achieve harmonious and coordinated development between economy and environment protection). Therefore, more benign coordinated development between higher education and environmental governance is possible if sufficient funding and support from the authorities are devoted. Former studies also prove that when fundings and policies are fully and reasonably applied, the coupling coordination development of regions will be achieved [74].

### 4.3 Coupling coordination degree (COU) prediction temporally and spatially

**Fig 8:**
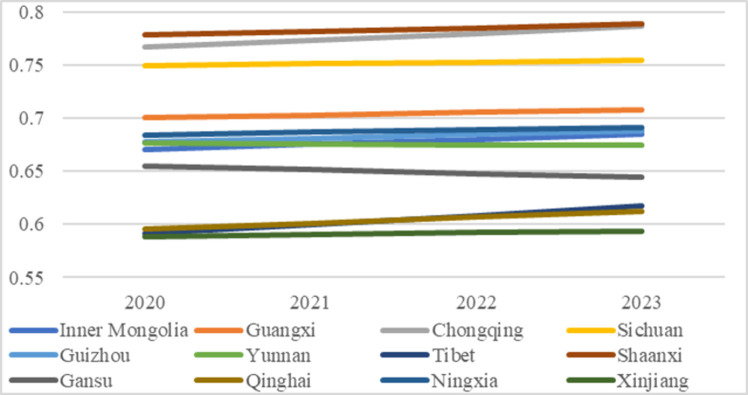
Temporal predictions of COU.

S4 Table exhibits the values of prediction tests and prediction results. All the regions passed the test and are available for predictions; the temporal changes are in Fig 8. We can find that the COU of the following years will remain stable with slight increases in most regions. In detail, 10 of the 12 regions will have apparent or slight increases, except Yunnan and Gansu, who will have slight declines in COU values. The increase of COU proves that when continuing to use the current strategies and devoting funds to higher education and environmental governance, most regions will maintain or increase the coupling coordination development. Besides, when evaluating the coupling coordination status with the “proportion” indicators, we can obtain more precise comparison results, which is beneficial for us to understand better the mutual interactions and growth between subsystems among different regions. However, some regions are facing potential decline, which signals policymakers to take urgent and specific actions to stop such decline immediately.

**Fig 9:**
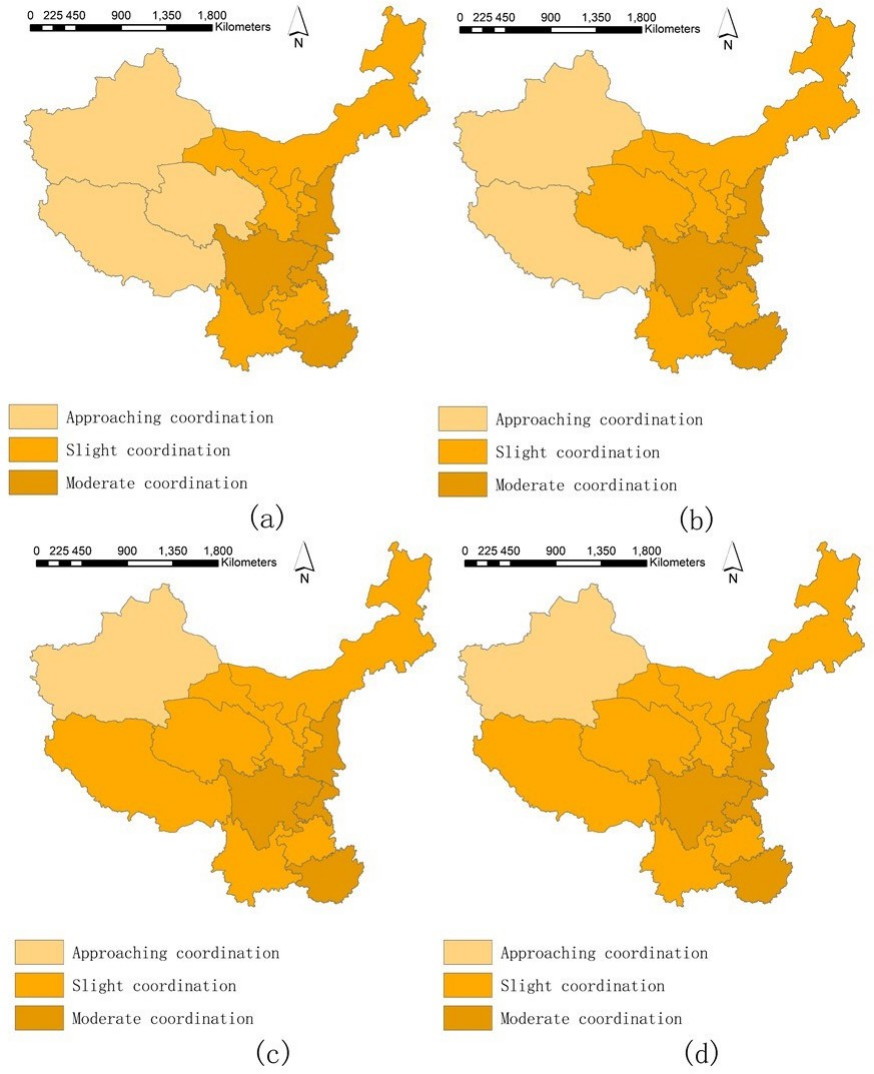
Spatial variation predictions of COU. (a-d) 2020–2023.

The predictions of the spatial variations of COU are in Fig 9. Though the gaps between the western regions (Xinjiang and Qinghai) and the rest regions still exist, it is glad to predict that such gaps will continue declining. Such decline is mainly because of the growth and up-gradation of COU in Tibet, so the differences between COU will gradually decrease in the upcoming years. Previous studies found that the performances or the coupling coordination status in other fields or subsystems in Tibet were relatively lagging [75, 76]. That was because those studies chose indicators measuring the “absolute quantity” rather than the comparable “proportion” or “per capita,” which was unfair, improper, and unprecise to assess the actual status of the fields or subsystems in Tibet, which is geographically large but demographically small. This prediction finding highlights that policies and actions should carefully consider population size differences and devote corresponding funds and resources so that regions can achieve highly efficient coordination development between subsystems.

## 5. Countermeasures

There are different performances of higher education and environmental governance from both temporal and spatial perspectives for different regions, and some regions witness the potential decline of the coupling coordination degrees; therefore, policymakers should propose specific while differentiated countermeasures to enhance the performances and the coupling coordination status between higher education and environmental governance. These countermeasures are also favorable references to other regions encountering similar situations.

For the western regions with relatively low performances and coupling coordination degrees of higher education and environmental governance (such as Xinjiang) and the regions with declining tendencies of the coordination status (such as Yunnan and Gansu), policymakers need to eliminate the obstructive factors and to prevent the decline of the coupling coordination degrees. There are several specific countermeasures.

1. The local policymakers should devote more budgets to the local higher education development and environmental governance; the money can be used in the teaching and research of the local higher education sector and waste treatment expenditures. Such accurate fiscal devotion will eliminate the obstacles of higher education input and environmental governance response actions and gradually prevent further decline.
2. Introduce special incentive policies to encourage better output of higher education and more peaceful state or responsive actions of environmental governance. The authorities can offer cash prizes, honors, and awards for extraordinary research projects, highly-recognized academic papers and publications, and those who actively protect the environment and participate in environmental impact assessment issues. The incentive policies will encourage as many people as possible to enhance their performances and the coordination between higher education and environmental governance with precise actions and results.

For regions with increasing performances and coupling coordination degrees (such as Tibet), it is essential to grasp the cherished opportunities to maintain such increasing tendencies and achieve better grades; policymakers can determine the critical competitive advantages or strengths and make the most of them. There are several suggestions to realize more harmonious coordination growth.

1. Make proper strategies and policies according to local conditions. Before deciding on the development pathways, policymakers should conduct an in-depth analysis of local core opportunities and strengths and invite third-party experts to optimize the pathways based on the local competitiveness. Adjusting measures to local conditions will enable the policymakers to develop the coupling coordination between subsystems efficiently on the right track.
2. Initiate cooperation programs with other regions or countries to explore new differentiated development approaches and to achieve better coupling coordination development. Regions are enabled to exchange resources they did not have; with the newly obtained resources, policymakers can propose new differentiated pathways to achieve better performances and a more benign coordination status. This policy will be beneficial for the regions to discover new opportunities and other key competitiveness so that the increased tendencies can be maintained.

For regions with relatively high performances and coupling coordination relations (such as Chongqing, Shaanxi, Guangxi, and Sichuan), policymakers can develop more and better methods to coordinate better higher education and environmental governance. There are some countermeasures.

1. Categorize different indicators and take differentiated approaches to enhance the values of indicators. It is better to maintain the status quo for the indicators that are difficult to further optimize in a short time (such as forest cover rate). It is better to take various actions for the indicators that can be further improved immediately (such as recruiting more professionals to evaluate environmental impact assessment documents and produce more academic publications). Differentiated treatment of different indicators helps policymakers enhance performance and coordination statuses efficiently.
2. Summarize the existing advanced experiences into theories and better apply these theories to explore new and innovative approaches in future development practices. Professionals are encouraged to participate in the whole process; the sublimation of practice into theory is helpful for policymakers to carry out practical innovation and enhance the coordination with new approaches.

Generally speaking, some effective countermeasures for national authorities can enhance the coordinated development in the country.

1. Initiate national strategies and laws to promote the coordinated development of higher education and environmental governance. The national strategies should be directive so local authorities can identify specific details suitable for local conditions, and the national laws should clarify the positive and negative lists. These will affect the local policymakers that their legal actions to enhance the local coordination performances will be supported and protected by the state power, and their illegal countermeasures will be strictly forbidden.
2. When evaluating the actual performances of each region, the national authority should consider the demographical differences; when allocating resources, the authority should consider proportions or “per capita” more than the absolute quantity. These will give policymakers more accurate insights into the coordination development between higher education and environmental governance.

## 6. Conclusions

This article selects the 12 western provincial regions as the case, explores their performances in higher education and environmental governance, assesses and predicts the coupling coordination degrees between higher education and environmental governance, and proposes countermeasures that are valuable references to other places with similar situations. Key findings are summarized below.

1. For the performances of both subsystems, the higher education subsystem is relatively more stable temporally; spatially, the distributions of the average performance grades of the two subsystems exist with apparent geographical differences.
2. For the coupling coordination degree, the degrees increase with mild fluctuations temporally; spatially, the grades of the coupling coordination degrees are upgrading, and the gaps among regions are gradually declining.
3. For the future tendencies of the coupling coordination status, temporally, the coupling coordination degrees of the following years will remain stable with slight increases in most regions; spatially, the gaps among regions will gradually decline.

There are several contributions to this study.

1. Academically, this study constructs a coupling coordination mechanism between higher education and environmental governance, establishes the aggregated evaluation index system to depict the coordination interaction modes, and proposes differentiated countermeasures. These enrich the theories of coupling coordination, higher education, and environmental governance and provide practical academic support for the western regions to achieve more peaceful higher education development and environmental governance.
2. Practically, this study provides applicable differentiated countermeasures based on the theoretical analysis and temporal-spatial comparisons; these measures are practical, feasible, and targeted and can provide significant support for policymakers to formulate follow-up policies and blueprints. These make the policy decision procedures more scientific, practical, and convincing.
3. Sustainably, this study explores the coupling coordination relations between higher education and environmental governance and obtains encouraging results that these two subsystems can achieve benign coordination interactions. It signals that social development and the environment can coexist harmoniously and promote each other; therefore, we can promote social growth without sacrificing the environment and promote environmental governance without giving up better social development. This insight further strengthens our determination to achieve sustainable development and environmental protection.

The limitations and future research agenda are as follows.

1. Some indices are excluded. For instance, indices "satisfaction with higher education" and "average concentrations of PM_2.5_" are neglected because the data are too challenging to obtain, though we admit they are essential actually; indices "number of students" and "total investment of waste treatments" are neglected because they focus on the absolute quantity rather than the proportion. The future research agenda would endeavor to collect more data to include those critical indices, carefully select some representative "absolute quantity" indices, and construct a comprehensive evaluation index system.
2. The study precision is limited. We only select annual data rather than quarterly or monthly data; besides, we only select the data of the provincial regions rather than the cities in these provincial regions. We admit that quarterly or monthly data of cities will significantly enhance the study precision and lead to a more detailed understanding of the coupling coordination status. The future research agenda would collect more detailed data (quarterly data of cities) to observe detailed temporal and spatial changes in the coupling coordination status.

## Supporting information

S1 Table (a, b) Performance of the higher education subsystem.
(ZIP)

S2 Table (a, b) Performance of the environmental governance subsystem.
(ZIP)

S3 Table (a, b) COU.
(ZIP)

S4 Table Predictions of COU. (DOCX)
