# Supplementary material for: Coupling coordination between higher education and environmental governance: Evidence of western China
Source: PLoS One. 2022 Aug 22;17(8):e0271994. doi: 10.1371/journal.pone.0271994 (PMC9394855; doi:10.1371/journal.pone.0271994)
Supplement: S1 Table — (a, b) Performance of the higher education subsystem. (ZIP) [file pone.0271994.s001.zip › S1(a)_Table.docx]

**S1(a) Table.** Performance of the Higher Education Subsystem.

|  | **2004** | **2005** | **2006** | **2007** | **2008** | **2009** | **2010** | **2011** | **2012** | **2013** |
| --- | --- | --- | --- | --- | --- | --- | --- | --- | --- | --- |
| **Inner Mongolia** | 0.3582 | 0.3858 | 0.3799 | 0.3694 | 0.3938 | 0.4368 | 0.3930 | 0.3031 | 0.3692 | 0.3563 |
| **Guangxi** | 0.4263 | 0.4704 | 0.4536 | 0.4410 | 0.4297 | 0.4697 | 0.4372 | 0.3640 | 0.3517 | 0.3790 |
| **Chongqing** | 0.5607 | 0.5915 | 0.5810 | 0.5880 | 0.5806 | 0.5748 | 0.6035 | 0.6204 | 0.5134 | 0.6153 |
| **Sichuan** | 0.5821 | 0.6137 | 0.6037 | 0.5777 | 0.6671 | 0.6211 | 0.6721 | 0.6724 | 0.6665 | 0.6108 |
| **Guizhou** | 0.2655 | 0.2948 | 0.3058 | 0.2914 | 0.2764 | 0.3584 | 0.3458 | 0.3403 | 0.3321 | 0.3421 |
| **Yunnan** | 0.4241 | 0.4237 | 0.4285 | 0.4380 | 0.4130 | 0.4452 | 0.4462 | 0.3939 | 0.3784 | 0.3877 |
| **Tibet** | 0.2786 | 0.2142 | 0.2871 | 0.3230 | 0.2493 | 0.2422 | 0.2358 | 0.2157 | 0.2602 | 0.1955 |
| **Shaanxi** | 0.7763 | 0.7705 | 0.6837 | 0.7302 | 0.6848 | 0.6389 | 0.6812 | 0.6545 | 0.6473 | 0.6854 |
| **Gansu** | 0.4709 | 0.5336 | 0.5351 | 0.4783 | 0.5162 | 0.5356 | 0.5099 | 0.4965 | 0.4857 | 0.5142 |
| **Qinghai** | 0.2551 | 0.3256 | 0.3533 | 0.3681 | 0.3303 | 0.3321 | 0.2960 | 0.3112 | 0.3261 | 0.3580 |
| **Ningxia** | 0.3290 | 0.4221 | 0.3419 | 0.3790 | 0.3776 | 0.4509 | 0.4012 | 0.3917 | 0.4576 | 0.4034 |
| **Xinjiang** | 0.2232 | 0.3236 | 0.2973 | 0.2776 | 0.3030 | 0.3759 | 0.2463 | 0.2597 | 0.3232 | 0.3148 |
