# Supplementary material for: Coupling coordination between higher education and environmental governance: Evidence of western China
Source: PLoS One. 2022 Aug 22;17(8):e0271994. doi: 10.1371/journal.pone.0271994 (PMC9394855; doi:10.1371/journal.pone.0271994)
Supplement: S1 Table — (a, b) Performance of the higher education subsystem. (ZIP) [file pone.0271994.s001.zip › S1(b)_Table.docx]

**S1(b) Table.** Performance of the Higher Education Subsystem.

|  | **2014** | **2015** | **2016** | **2017** | **2018** | **2019** | **Average value** | **Average grade** |
| --- | --- | --- | --- | --- | --- | --- | --- | --- |
| **Inner Mongolia** | 0.3482 | 0.3841 | 0.3991 | 0.4230 | 0.3648 | 0.2877 | 0.3720 | Ordinary |
| **Guangxi** | 0.3604 | 0.3623 | 0.4180 | 0.3729 | 0.4259 | 0.3848 | 0.4092 | Fair |
| **Chongqing** | 0.5882 | 0.5644 | 0.6163 | 0.6608 | 0.7346 | 0.7068 | 0.6063 | Good |
| **Sichuan** | 0.5967 | 0.6112 | 0.6482 | 0.5544 | 0.5956 | 0.6095 | 0.6189 | Good |
| **Guizhou** | 0.3462 | 0.3874 | 0.4072 | 0.3791 | 0.4542 | 0.4096 | 0.3460 | Ordinary |
| **Yunnan** | 0.3221 | 0.3802 | 0.4473 | 0.4242 | 0.3462 | 0.3499 | 0.4030 | Fair |
| **Tibet** | 0.2692 | 0.1844 | 0.3354 | 0.1808 | 0.3211 | 0.2895 | 0.2551 | Ordinary |
| **Shaanxi** | 0.6903 | 0.7558 | 0.7509 | 0.6150 | 0.6305 | 0.6533 | 0.6905 | Good |
| **Gansu** | 0.4500 | 0.4926 | 0.5020 | 0.4699 | 0.3973 | 0.3811 | 0.4856 | Fair |
| **Qinghai** | 0.4401 | 0.4543 | 0.4104 | 0.4455 | 0.4483 | 0.4367 | 0.3682 | Ordinary |
| **Ningxia** | 0.3518 | 0.4395 | 0.4376 | 0.4502 | 0.6104 | 0.4860 | 0.4206 | Fair |
| **Xinjiang** | 0.2873 | 0.2552 | 0.3659 | 0.3489 | 0.2671 | 0.2421 | 0.2944 | Ordinary |
