# Supplementary material for: Coupling coordination between higher education and environmental governance: Evidence of western China
Source: PLoS One. 2022 Aug 22;17(8):e0271994. doi: 10.1371/journal.pone.0271994 (PMC9394855; doi:10.1371/journal.pone.0271994)
Supplement: S2 Table — (a, b) Performance of the environmental governance subsystem. (ZIP) [file pone.0271994.s002.zip › S2(a)_Table.docx]

**S2(a) Table.** Performance of the Environmental Governance Subsystem.

|  | **2004** | **2005** | **2006** | **2007** | **2008** | **2009** | **2010** | **2011** | **2012** | **2013** |
| --- | --- | --- | --- | --- | --- | --- | --- | --- | --- | --- |
| **Inner Mongolia** | 0.4205 | 0.2932 | 0.3096 | 0.4000 | 0.3891 | 0.3858 | 0.3974 | 0.3915 | 0.4971 | 0.4000 |
| **Guangxi** | 0.4548 | 0.3577 | 0.4718 | 0.4530 | 0.4589 | 0.5144 | 0.4772 | 0.6682 | 0.6200 | 0.4515 |
| **Chongqing** | 0.3295 | 0.3453 | 0.5790 | 0.3679 | 0.3737 | 0.4506 | 0.3756 | 0.4773 | 0.3430 | 0.3677 |
| **Sichuan** | 0.7377 | 0.5398 | 0.5002 | 0.4334 | 0.4376 | 0.4273 | 0.3660 | 0.4349 | 0.5520 | 0.4023 |
| **Guizhou** | 0.4707 | 0.5474 | 0.5409 | 0.5750 | 0.5591 | 0.4995 | 0.6401 | 0.4973 | 0.5243 | 0.3991 |
| **Yunnan** | 0.5060 | 0.5457 | 0.4975 | 0.5109 | 0.5197 | 0.5888 | 0.4700 | 0.5410 | 0.5131 | 0.4766 |
| **Tibet** | 0.2414 | 0.2095 | 0.2423 | 0.2408 | 0.2627 | 0.2591 | 0.2497 | 0.3222 | 0.2214 | 0.3192 |
| **Shaanxi** | 0.5540 | 0.3549 | 0.4944 | 0.4152 | 0.4230 | 0.4237 | 0.4431 | 0.5167 | 0.4229 | 0.4511 |
| **Gansu** | 0.4437 | 0.3835 | 0.3967 | 0.5567 | 0.5443 | 0.4834 | 0.4739 | 0.4704 | 0.3225 | 0.6137 |
| **Qinghai** | 0.2682 | 0.1955 | 0.2300 | 0.2390 | 0.2436 | 0.2776 | 0.2605 | 0.2570 | 0.2264 | 0.2175 |
| **Ningxia** | 0.3829 | 0.6762 | 0.3828 | 0.6244 | 0.6039 | 0.4822 | 0.3166 | 0.3559 | 0.2312 | 0.3743 |
| **Xinjiang** | 0.5362 | 0.3448 | 0.3120 | 0.3469 | 0.3609 | 0.3663 | 0.3288 | 0.3604 | 0.3116 | 0.3393 |
