# Supplementary material for: Coupling coordination between higher education and environmental governance: Evidence of western China
Source: PLoS One. 2022 Aug 22;17(8):e0271994. doi: 10.1371/journal.pone.0271994 (PMC9394855; doi:10.1371/journal.pone.0271994)
Supplement: S2 Table — (a, b) Performance of the environmental governance subsystem. (ZIP) [file pone.0271994.s002.zip › S2(b)_Table.docx]

**S2(b) Table.** Performance of the Environmental Governance Subsystem.

|  | **2014** | **2015** | **2016** | **2017** | **2018** | **2019** | **Average value** | **Average grade** |
| --- | --- | --- | --- | --- | --- | --- | --- | --- |
| **Inner Mongolia** | 0.5698 | 0.4679 | 0.5170 | 0.5098 | 0.5059 | 0.5075 | 0.4351 | Fair |
| **Guangxi** | 0.5277 | 0.4913 | 0.6703 | 0.5646 | 0.6186 | 0.6397 | 0.5275 | Fair |
| **Chongqing** | 0.4400 | 0.3971 | 0.5035 | 0.4847 | 0.5537 | 0.5735 | 0.4351 | Fair |
| **Sichuan** | 0.4802 | 0.4336 | 0.4850 | 0.5103 | 0.5857 | 0.5839 | 0.4944 | Fair |
| **Guizhou** | 0.5022 | 0.4248 | 0.5027 | 0.4692 | 0.5470 | 0.5631 | 0.5164 | Fair |
| **Yunnan** | 0.5684 | 0.5457 | 0.6123 | 0.5370 | 0.5780 | 0.6020 | 0.5383 | Fair |
| **Tibet** | 0.3912 | 0.3579 | 0.4215 | 0.3948 | 0.4400 | 0.4385 | 0.3133 | Ordinary |
| **Shaanxi** | 0.5181 | 0.4320 | 0.5131 | 0.4892 | 0.5784 | 0.5929 | 0.4764 | Fair |
| **Gansu** | 0.4248 | 0.3747 | 0.3760 | 0.4749 | 0.4519 | 0.4465 | 0.4524 | Fair |
| **Qinghai** | 0.2231 | 0.2827 | 0.3668 | 0.2891 | 0.2360 | 0.2280 | 0.2526 | Ordinary |
| **Ningxia** | 0.5245 | 0.6035 | 0.4968 | 0.5468 | 0.4507 | 0.4515 | 0.4690 | Fair |
| **Xinjiang** | 0.4570 | 0.3752 | 0.4127 | 0.3747 | 0.4202 | 0.4215 | 0.3793 | Ordinary |
