# Supplementary material for: Coupling coordination between higher education and environmental governance: Evidence of western China
Source: PLoS One. 2022 Aug 22;17(8):e0271994. doi: 10.1371/journal.pone.0271994 (PMC9394855; doi:10.1371/journal.pone.0271994)
Supplement: S3 Table — (a, b) COU. (ZIP) [file pone.0271994.s003.zip › S3(a)_Table.docx]

**S3(a) Table.** COU.

|  | **2004** | **2005** | **2006** | **2007** | **2008** | **2009** | **2010** | **2011** |
| --- | --- | --- | --- | --- | --- | --- | --- | --- |
| **Inner Mongolia** | 0.6230 | 0.5799 | 0.5856 | 0.6200 | 0.6257 | 0.6407 | 0.6286 | 0.5869 |
| **Guangxi** | 0.6635 | 0.6404 | 0.6801 | 0.6686 | 0.6664 | 0.7011 | 0.6758 | 0.7023 |
| **Chongqing** | 0.6556 | 0.6722 | 0.7616 | 0.6820 | 0.6825 | 0.7134 | 0.6900 | 0.7377 |
| **Sichuan** | 0.8095 | 0.7587 | 0.7413 | 0.7074 | 0.7350 | 0.7178 | 0.7042 | 0.7354 |
| **Guizhou** | 0.5946 | 0.6338 | 0.6377 | 0.6398 | 0.6270 | 0.6504 | 0.6859 | 0.6414 |
| **Yunnan** | 0.6806 | 0.6935 | 0.6795 | 0.6878 | 0.6807 | 0.7155 | 0.6767 | 0.6794 |
| **Tibet** | 0.5093 | 0.4602 | 0.5136 | 0.5281 | 0.5059 | 0.5005 | 0.4926 | 0.5134 |
| **Shaanxi** | 0.8098 | 0.7232 | 0.7625 | 0.7420 | 0.7336 | 0.7213 | 0.7412 | 0.7626 |
| **Gansu** | 0.6761 | 0.6726 | 0.6788 | 0.7183 | 0.7281 | 0.7133 | 0.7011 | 0.6952 |
| **Qinghai** | 0.5115 | 0.5023 | 0.5339 | 0.5446 | 0.5326 | 0.5510 | 0.5270 | 0.5318 |
| **Ningxia** | 0.5958 | 0.7310 | 0.6015 | 0.6975 | 0.6911 | 0.6828 | 0.5970 | 0.6111 |
| **Xinjiang** | 0.5882 | 0.5780 | 0.5519 | 0.5571 | 0.5750 | 0.6092 | 0.5335 | 0.5531 |
