# Supplementary material for: Coupling coordination between higher education and environmental governance: Evidence of western China
Source: PLoS One. 2022 Aug 22;17(8):e0271994. doi: 10.1371/journal.pone.0271994 (PMC9394855; doi:10.1371/journal.pone.0271994)
Supplement: S3 Table — (a, b) COU. (ZIP) [file pone.0271994.s003.zip › S3(b)_Table.docx]

**S3(b) Table.** COU.

|  | **2012** | **2013** | **2014** | **2015** | **2016** | **2017** | **2018** | **2019** |
| --- | --- | --- | --- | --- | --- | --- | --- | --- |
| **Inner Mongolia** | 0.6545 | 0.6144 | 0.6674 | 0.6511 | 0.6740 | 0.6815 | 0.6554 | 0.6181 |
| **Guangxi** | 0.6833 | 0.6432 | 0.6604 | 0.6495 | 0.7275 | 0.6774 | 0.7165 | 0.7044 |
| **Chongqing** | 0.6478 | 0.6897 | 0.7132 | 0.6881 | 0.7464 | 0.7523 | 0.7986 | 0.7979 |
| **Sichuan** | 0.7788 | 0.7041 | 0.7316 | 0.7175 | 0.7488 | 0.7293 | 0.7685 | 0.7724 |
| **Guizhou** | 0.6460 | 0.6079 | 0.6458 | 0.6369 | 0.6726 | 0.6494 | 0.7060 | 0.6930 |
| **Yunnan** | 0.6638 | 0.6556 | 0.6541 | 0.6749 | 0.7234 | 0.6909 | 0.6688 | 0.6775 |
| **Tibet** | 0.4899 | 0.4998 | 0.5697 | 0.5069 | 0.6132 | 0.5169 | 0.6131 | 0.5969 |
| **Shaanxi** | 0.7233 | 0.7457 | 0.7734 | 0.7559 | 0.7878 | 0.7406 | 0.7771 | 0.7889 |
| **Gansu** | 0.6291 | 0.7495 | 0.6613 | 0.6554 | 0.6591 | 0.6873 | 0.6509 | 0.6423 |
| **Qinghai** | 0.5213 | 0.5283 | 0.5598 | 0.5986 | 0.6229 | 0.5991 | 0.5703 | 0.5617 |
| **Ningxia** | 0.5703 | 0.6234 | 0.6554 | 0.7176 | 0.6828 | 0.7044 | 0.7242 | 0.6844 |
| **Xinjiang** | 0.5633 | 0.5717 | 0.6020 | 0.5563 | 0.6233 | 0.6013 | 0.5788 | 0.5652 |
