# Supplementary material for: Coupling coordination between higher education and environmental governance: Evidence of western China
Source: PLoS One. 2022 Aug 22;17(8):e0271994. doi: 10.1371/journal.pone.0271994 (PMC9394855; doi:10.1371/journal.pone.0271994)
Supplement: S4 Table — (DOCX) [file pone.0271994.s004.docx]

**S4 Table.** Predictions of COU.

|  | **a** | **EP** | **2020** | **2021** | **2022** | **2023** |
| --- | --- | --- | --- | --- | --- | --- |
| **Inner Mongolia** | -0.0074 | 0.7500 | 0.6703 | 0.6753 | 0.6803 | 0.6853 |
| **Guangxi** | -0.0037 | 0.6875 | 0.7004 | 0.7030 | 0.7056 | 0.7083 |
| **Chongqing** | -0.0160 | 0.6875 | 0.7672 | 0.7736 | 0.7801 | 0.7866 |
| **Sichuan** | -0.0022 | 0.6875 | 0.7497 | 0.7514 | 0.7531 | 0.7547 |
| **Guizhou** | -0.0050 | 0.6875 | 0.6780 | 0.6813 | 0.6848 | 0.6882 |
| **Yunnan** | 0.0020 | 0.7500 | 0.6763 | 0.6757 | 0.6750 | 0.6744 |
| **Tibet** | -0.0144 | 0.8125 | 0.5912 | 0.5998 | 0.6085 | 0.6173 |
| **Shaanxi** | -0.0016 | 0.7500 | 0.7784 | 0.7818 | 0.7852 | 0.7886 |
| **Gansu** | 0.0052 | 0.8125 | 0.6548 | 0.6514 | 0.6480 | 0.6446 |
| **Qinghai** | -0.0095 | 0.6250 | 0.5955 | 0.6012 | 0.6070 | 0.6128 |
| **Ningxia** | -0.0036 | 0.5625 | 0.6843 | 0.6868 | 0.6892 | 0.6917 |
| **Xinjiang** | -0.0030 | 0.7500 | 0.5886 | 0.5904 | 0.5921 | 0.5939 |
